# Cases from the Royal Hospital for Children and Women, London

**Published:** 1901-09-21

**Authors:** C. O. Hawthorne

**Affiliations:** Assistant Physician to the Hospital, Physician to the Central London Ophthalmic Hospital, and Examiner in Materia Medica and Therapeutics in the University of Edinburgh


### HOSPITAL CLINICS AND MEDICAL PROGRESS.

### CASES FROM THE ROYAL HOSPITAL FOR CHILDREN AND WOMEN, LONDON.

By C. O. HAWTHORNE, M.D., M.R.C.P., Assistant Physician to the Hospital, Physician to the Central London Ophthalmic Hospital, and Examiner in Materia Medica and Therapeutics in the University of Edinburgh.

### A CASE OF DIPHTHERIA.

THIS patient is a boy of 10 years. His mother tells us that he was in his usual health until yesterday, when he began to complain of a sore throat. On examination the right tonsil is seen to be unduly large and red, and on its surface is a single dirty yellowish-white patch. The question is at once raised—Is this a case of diphtheria? I hardly think anyone can feel quite certain here from a mere inspection of the throat. The fact that the patch is single favours diphtheria rather than follicular tonsillitis, but it might well be due to a coalescence of several of the pustular points of the latter disease. Had the patch been present on the soft palate instead of on the tonsil there could have been little doubt that the case was one of diphtheria. But as it is, the mere inspection of the throat leaves us in doubt, and we must seek other evidence if we are to make a confident diagnosis. It is obvious that as we have here and now to decide whether the boy is to be sent away to hospital, or allowed to return to his home, we cannot wait for a bacteriological examination. Well, we will take the boy's temperature. It is barely 99·5° Fahr. That is a fact strongly in favour of a diagnosis of diphtheria. Acute tonsillitis usually produces high fever from the outset, whilst in diphtheria the temperature for the first few days is often normal or only slightly raised. Then the boy's pulse is 130. Of course, just now he is excited and alarmed. But all the same we cannot but remember that the rate of the pulse is altogether out of proportion to the height of the temperature, and that this is the usual condition in early diphtheria. Next we observe that the glands near the angle of the jaw on the right side are enlarged and tender. This also is, as an early occurrence, a fact favouring a diagnosis of diphtheria rather than of follicular tonsillitis. The knee-jerks, I find, are present and distinct. Had they been absent there could have been no doubt of the nature of the case. It is not only when diphtheritic paralysis develops that the patient loses his knee-jerks. Sometimes quite at the outset of the disease this loss occurs, and when this is the case it is a valuable aid to diagnosis. Next we examine the urine, and we find albumen in considerable amount. That, I think, settles the diagnosis. Albuminuria is not a feature of tonsillitis, whilst it is fairly common in the early stage of an attack of diphtheria. The sooner the boy is put to bed and kept there the better the chance of avoiding the heart failure which is one of the chief dangers of diphtheria, and the sooner he is isolated the better for his relations and neighbours. The house surgeon will therefore notify the case, and the boy will be sent to hospital, where he will no doubt be put to bed, given abundant fluid nourishment, and brought under the influence of antitoxin. The case shows very well how diphtheria at the outset often causes little discomfort or pain. Indeed a patient who has much pain, considerable difficulty in swallowing, and cannot open his mouth widely, is far more likely to be the subject of tonsillitis rather than of diphtheria. The case also shows how, when the appearances in the throat are of uncertain interpretation, a diagnosis may be often made without waiting for a bacteriological report.

### GREY HAIRS IN ONE EYEBROW.

Mrs. L., *æt.* 35 years, wants to know if we can do anything for the condition of her right eyebrow, nearly all the hairs in which are grey. The hairs of the scalp, with the exception of a patch in the right temporal region, and those of the left eyebrow, as well as the right eyelashes, are all very dark, and natural in appearance. She says that about fifteen years ago she received a severe mental shock which caused her to have "a fit." The next day both she herself and her mother noticed the grey hairs in her right eyebrow and on the right side of her head. Since that date there has been no change. Her general health has been quite good, and no new grey hairs have made their appearance. That is her story, and I see no reason to doubt its accuracy. Greyness of the hair must mean some change in its nutrition, and is known to be associated with the presence of air in the medulla or pith. It is reasonable to believe that this nutritive change may be induced through the agency of the nervous system. And very probably it may be induced with great suddenness. Apart from the historic instances in which the hair is reported to have turned grey in a single night, there are some authorities who hold that hair when it turns grey always does so suddenly. The usual thing, according to this view, is that each hair turns grey suddenly but the change only affects one or a few hairs at one and the same time, and consequently, as far as the whole scalp is concerned, the change is a gradual one. In the cases in which the whole hair has become grey in a single night the peculiarity is, not that individual hairs have changed with unusual swiftness, but that all the hairs have undergone the change at one and the same time—they have all, so to speak, selected the same night on which to become grey. It does not seem possible to help our patient except by telling her that if her happiness or success depends upon the condition of her right eyebrow she must dye it. I hope that it is not unprofessional to give that advice. At all events, so far as I know, there is no other solution of the difficulty.

### HEADACHE DUE TO ASTIGMATISM.

This lad first came to the hospital two months ago. He complained of attacks of severe headache. His mother told us that he was a very successful lad at school and was very fond of reading, and that with the exception of the headaches he had no other ground of complaint. When examined there was no evidence of any disease in the chest or abdomen. There was no sign of organic nervous disease. The urine was free from albumen. There was no optic neuritis. It is the only safe plan in cases of headache to make sure, by a complete examination, of the absence of organic disease. Headache may be due to various causes, some of them of a most serious nature. It may for example be the only complaint in a case of brain tumour. Hence the importance of an ophthalmoscopic examination. But in our present patient physical examination failed to discover any abnormal fact suggesting visceral disease. The ophthalmoscopic examination did, however, tell us that the boy's refraction was at fault, and when we put him to read the test types we found his vision seriously defective. His refraction was therefore estimated and he was found to have two dioptres of simple hypermetropic astigmatism. He was ordered appropriate glasses, and now, as he tells us himself, he never has headaches, and he reads the lowest line of type on the test cards. By testing his vision we discovered the cause of the headache, and spectacles have done for him what physic would never have accomplished.

Thickening of the Tibiæ in Hereditary Syphilis.

Nellie R., *æt.* 8 years, is brought here by her mother because she is said to be getting weak and losing flesh. Her appetite is poor, but there is no vomiting, and the bowels act daily. The child's complaints are, of course, frequently heard in out-patient hospital practice, and in the majority of instances depend on insufficient or injudicious food. But as far as possible I like to thoroughly examine every patient, at least at the first visit, therefore the nurse will strip the child for us. There seems at first sight nothing very remarkable to be discovered. The general nutrition is perhaps below par, but there is no physical evidence of disease in the chest or abdomen, unless it be that the spleen is rather large, so that its lower end can be palpated just below the margin of the ribs. The mother, however, asks us to look at the child's legs which she says are getting "bowed." Now we observe a fact of great interest. The inner (subcutaneous) surface of each tibia is prominent, convex, and rounded, so that at first sight it looks as though the legs were curved inwards. But, as we see when the girl stands upright, this is not really the case, for the limbs are quite straight. When the mother speaks of the legs as being "bowed" she probably has in her mind the notion that the condition is due to rickets, and this is a mistake which is commonly made. The condition is not due to rickets, but to a much more serious cause, viz., inherited syphilis. Sometimes there is associated with it effusion into the joints, as, *e.g.*, the knees, but that is not the case here. It may seem rather a rash thing to make so serious a diagnosis on the basis of a single symptom. Possibly it is, yet I have not much doubt that the diagnosis is correct. The condition is certainly not rickets. The deformity of the legs is quite different from a rachitic deformity, and there is no evidence of rickets in the bones of the head, chest, or upper limbs. And further, that disease is not very likely to commence at 7 or 8 years of age. On the other hand, it is quite true that the child has not the physiognomy of hereditary syphilis, nor is there any other evidence of that disease, unless it be the slight enlargement of the spleen. The mother tells us she has no other living children, so that we cannot adopt the plan of endeavouring to confirm the diagnosis by examining other members of the family. That is a very useful measure, especially in cases where a child is brought with a single fact suggesting hereditary specific disease. It is not, however, available to us here. Yet the family record could hardly be more significant, for we learn that before the birth of our present patient there were three pregnancies, and that each terminated between the seventh and eighth month with the birth of a dead fœtus. The mother herself has enjoyed good health during the whole of her married life. This thickening of the inner surface of the tibia is worth noting. It is not perhaps a very frequent expression of hereditary syphilis, but when it occurs it is very significant. Of course it depends on a chronic periostitis. For treatment we must by suitable food, by cod-liver oil, and by hygienic measures endeavour to improve the child's general health. In addition, I think we had better order a grain of grey powder twice a day. Whether it will do anything to prevent the development of other symptoms, to which, of course, the child is liable, is perhaps uncertain. But it is at least in the right direction and can do the child no harm.

